# Supplementary material for: Ovarian Pregnancy: 2 Case Reports and a Systematic Review
Source: J Clin Med. 2023 Feb 1;12(3):1138. doi: 10.3390/jcm12031138 (PMC9917991; doi:10.3390/jcm12031138)
Supplement: Supplementary file 1 [file jcm-12-01138-s001.zip › jcm-2148447-supplementary.pdf]

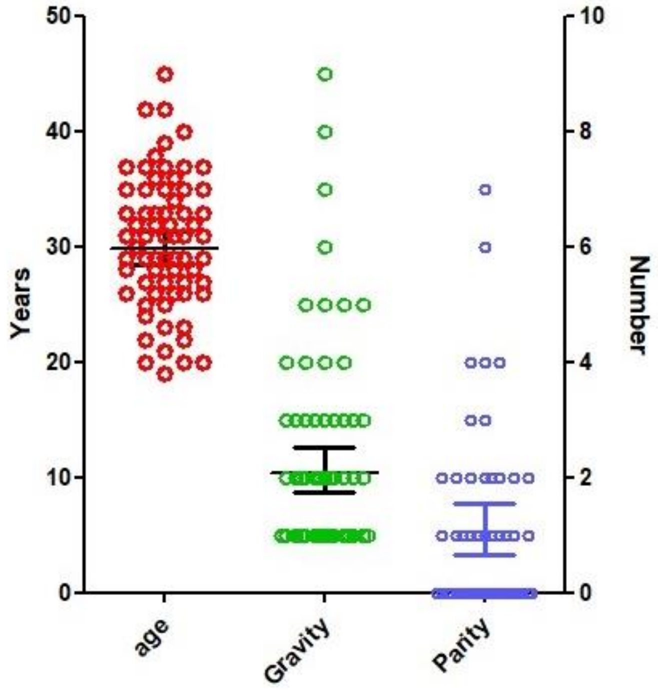

Figure S1: Age, parity and gravity of women with an ovarian pregnancy. Geometric means and confidence limits are indicated.
